# Positive Allosteric Modulators of Trk Receptors for the Treatment of Alzheimer’s Disease

**DOI:** 10.3390/ph17080997

**Published:** 2024-07-28

**Authors:** Pontus Forsell, Cristina Parrado Fernández, Boel Nilsson, Johan Sandin, Gunnar Nordvall, Märta Segerdahl

**Affiliations:** 1 AlzeCure Pharma AB, Hälsovägen 7, 141 57 Huddinge, Sweden; cristina.parrado@alzecurepharma.com (C.P.F.); boel.nilsson@alzecurepharma.com (B.N.); johan.sandin@alzecurepharma.com (J.S.); gunnar.nordvall@alzecurepharma.com (G.N.); marta.segerdahl@alzecurepharma.com (M.S.); 2 Department of Neurobiology, Care Sciences and Society, Karolinska Institutet, Alfred Nobels allé 23, 141 52 Huddinge, Sweden

**Keywords:** neurotrophins, brain-derived neurotrophic factor (BDNF), nerve growth factor (NGF), Alzheimer’s disease, neurodegeneration

## Abstract

Neurotrophins are important regulators of neuronal and non-neuronal functions. As such, the neurotrophins and their receptors, the tropomyosin receptor kinase (Trk) family of receptor tyrosine kinases, has attracted intense research interest and their role in multiple diseases including Alzheimer’s disease has been described. Attempts to administer neurotrophins to patients have been reported, but the clinical trials have so far have been hampered by side effects or a lack of clear efficacy. Thus, much of the focus during recent years has been on identifying small molecules acting as agonists or positive allosteric modulators (PAMs) of Trk receptors. Two examples of successful discovery and development of PAMs are the TrkA-PAM E2511 and the pan-Trk PAM ACD856. E2511 has been reported to have disease-modifying effects in preclinical models, whereas ACD856 demonstrates both a symptomatic and a disease-modifying effect in preclinical models. Both molecules have reached the stage of clinical development and were reported to be safe and well tolerated in clinical phase 1 studies, albeit with different pharmacokinetic profiles. These two emerging small molecules are interesting examples of possible novel symptomatic and disease-modifying treatments that could complement the existing anti-amyloid monoclonal antibodies for the treatment of Alzheimer’s disease. This review aims to present the concept of positive allosteric modulators of the Trk receptors as a novel future treatment option for Alzheimer’s disease and other neurodegenerative and cognitive disorders, and the current preclinical and clinical data supporting this new concept. Preclinical data indicate dual mechanisms, not only as cognitive enhancers, but also a tentative neurorestorative function.

## 1. Introduction

**Figure 1:**
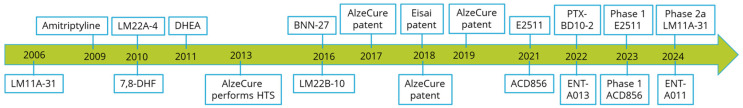
A schematic timeline showing a selection of major achievements in the identification or development of positive modulators of neurotrophin signaling including publication of scientific articles, submission of patent applications or performed clinical trials.

Recent advancements and breakthroughs in the diagnostics, treatment, and monitoring of Alzheimer’s disease (AD) have spurred the interest in novel therapeutics targeting this devastating disease. The results from late-stage clinical trials with monoclonal anti-amyloid antibodies and the approval of lecanemab and donanemab are encouraging, and these antibodies have indeed demonstrated very high clearance of the amyloid plaques and a reduction in the disease progression, as defined by slowing cognitive decline, in approximately 25–30% in patients with early AD [1,2,3,4]. The reduction in amyloid pathology by donanemab translates into a delay of disease progression of approximately 5.3 months [5]. Despite the success of anti-amyloid antibodies in clearing amyloid pathology, the modest reduction in cognitive decline suggests that there is ample opportunity for new therapeutics targeting non-amyloid pathways. By targeting other molecular pathways that are more directly correlated with cognitive function than the amyloid cascade, e.g., synaptic plasticity and neuronal dysfunction, such therapeutics could be an efficient add-on treatment to anti-amyloid antibodies, especially if it were to combine symptomatic effects with disease-modifying effects. The introduction of anti-amyloid antibodies has brought forth a first generation of disease-modifying treatments for AD. Several approaches are now being developed to generate a second generation of disease-modifying and/or symptomatic treatments for AD. One such approach is based upon enhancement of neurotrophin receptor signaling, a key element in neuronal function and brain health. The aim of this review is to discuss the advancements in the development of positive allosteric modulators of neurotrophin receptors. In this review, we will provide a brief overview of the current understanding of neurotrophins and their receptors and how they exert their effects, and thereafter discuss the recent development of novel small-molecule positive allosteric modulators. Although there have been several attempts to identify molecules that enhance signaling of neurotrophins—such as natural products, small-molecule peptidomimetics, and receptor agonists—the objective of this review is to focus on the development of novel compounds acting as positive allosteric modulators in clinical development within the field of neurotrophin receptor allostery, with special focus on diseases related to the central nervous system (CNS) such as AD. Considering the current results on cognitive decline with monoclonal anti-amyloid antibodies in patients with mild AD, we think it is of high relevance to summarize the recent literature on an additional mechanism for the treatment of AD, i.e., small-molecule positive allosteric modulators within the field of neurotrophins. We have chosen to highlight the ongoing clinical efforts with small molecules that potentially could complement the anti-amyloid approach in the treatment of Alzheimer’s disease in the near future. In summary, we discuss herein compounds described as modulators of neurotrophin receptors with a focus on Trk receptors and the most recent and important findings in the identification of novel molecules targeting the Trk family of receptors as depicted in Figure 1.

## 2. Neurotrophins and the Trk Receptor Family

The functional effects of the neurotrophins (NTs) have long been known and the seminal paper by Rita Levi-Montalcini [6] described the effects of nerve growth factor (NGF), the first discovered member of the neurotrophin family. The gene for NGF was later identified independently by two different groups in 1983 [7,8]. Apart from NGF, the mammalian family of neurotrophins includes brain-derived neurotrophic factor (BDNF) [9,10], NT-3 [11,12], and NT4/5 [13,14,15], which bind to the cognate tropomyosin receptor kinase (Trk) family of receptor tyrosine kinases, including TrkA [16], TrkB [17], and TrkC [18] as well as to a common receptor p75NTR, belonging to the tumor necrosis factor receptor superfamily [19].

### 2.1. Brain Expression Pattern and Function of the Neurotrophins and Their Receptors 

NTs, including NGF, BDNF, and NT-3 through NT-7, have long been known to be found in a wide variety of vertebrate species including mammals, birds, reptiles, amphibians, and fishes [20]. It is suggested that the family of NTs and their cognate Trk receptors evolved early in vertebrates. Indeed, whole genome sequencing projects reveal the presence of neurotrophin-like proteins in invertebrate species previously thought to lack NTs, thus implying that the neurotrophin system evolved very early in the animal kingdom [21,22]. Interestingly, NGF-like genes have also been identified in avian viruses including fowlpox and canarypox [20]. The enigmatic role of NTs in avian viruses might be explained by an effect of viral NGF-like proteins on host-related mechanisms leading to increased viral survival or replication.

**Figure 2:**
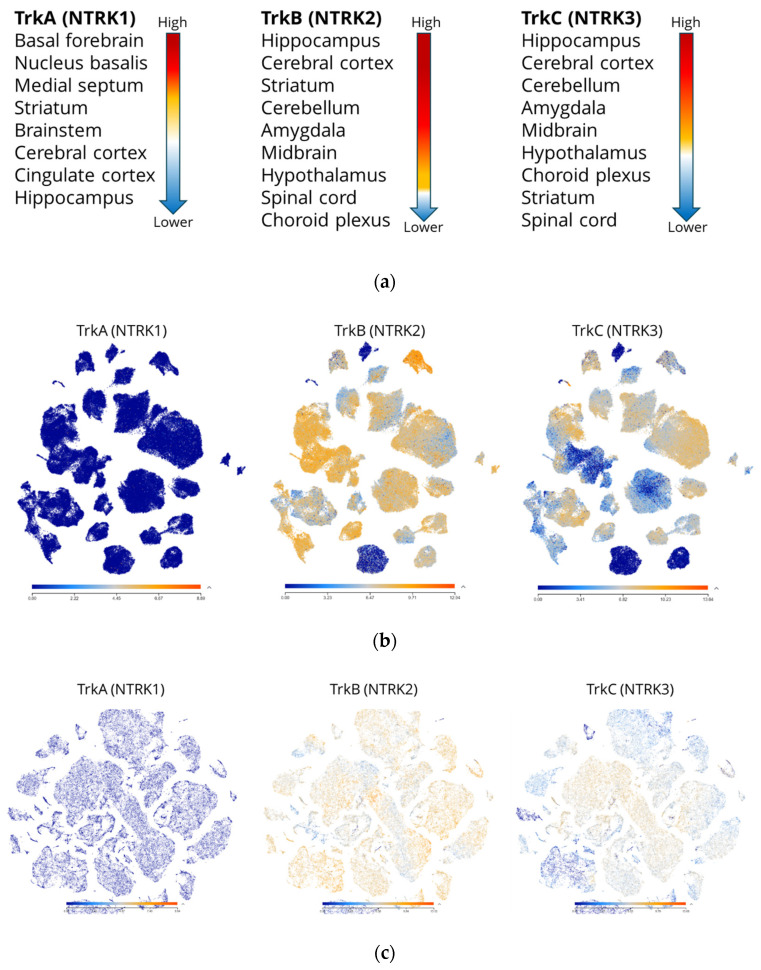
(**a**) A schematic representation of Trk receptor expression summarized from [21,24,25,26,27] and the Human Protein Atlas initiative (https://www.proteinatlas.org, accessed on 16 June 2024) where red indicates high levels and blue indicates low levels of expression. Figures (**b**,**c**) are RNAseq data from Allen Brain Map, Allen Institute for Brain Science; Human multiple cortical areas—SMART-seq (https://celltypes.brain-map.org/rnaseq, accessed on 16 June 2024). (**b**) This data set includes single-nucleus transcriptomes from 49,495 nuclei across multiple human cortical areas. Individual layers of cortex were dissected from tissues covering the middle temporal gyrus. (**c**) The data set includes single-cell transcriptomes from 76,533 total cells derived from two post-mortem human brain specimens in the primary motor cortex.

The expression of NTs and their receptors is extensively characterized in the human central and peripheral nervous system [23,24,25,26]. Generally, TrkB and TrkC exhibit a more widespread expression pattern than TrkA. Figure 2a summarizes the findings in previous reports [21,24,25,26,27], whereas Figure 2b and c show the expression pattern of Trk receptors in different regions of the huma brain, demonstrating the broad expression profile of TrkB and TrkC in certain regions of the brain.

Apart from binding to Trk receptors, NTs and their pro-forms also bind to the p75NTR receptor [19]. Signaling via p75NTR has been shown to have pleiotropic effects in multiple cell types [28]. Apart from the more commonly described effects such as regulation of apoptosis, pro- [29] and anti-inflammatory [30] effects have also been ascribed to p75NTR-signaling. The plethora of spatial, temporal, and receptor type-specific signaling is likely to contribute to the wealth of physiological and pathological responses attributed to NT signaling, including both neuronal and non-neuronal functions. The NTs and Trk receptors are undoubtedly important for the development and maintenance of both the peripheral and central nervous systems in vertebrates. NT signaling elicits multiple biological effects, including neuronal plasticity and cognitive function, mitochondrial function, peripheral bioenergetics, proliferation, and differentiation, as well as anti- and pro-inflammatory responses [31,32]. NTs have long-term effects by regulating changes in gene expression [33,34] as well as short-term effects on the phosphorylation state of specific adaptor proteins [35]. The most well-studied downstream effector proteins of Trk receptors includes SHC1, PI3K, PLCγ1, ERK1/2, and AKT. Through the action of these effector proteins, Trk receptors modulate synaptic function by regulating the expression of ion channels, membrane potential, and synaptic plasticity [36]. These different levels of regulation involve both ligand-gated ion channels [35,37] and voltage-gated sodium channels (VGSC) [38].

Given the broad function of NTs, it is not surprising that several diseases are associated with altered levels of NTs or disrupted receptor signaling. A number of well-known diseases such as AD [39], neuropsychiatric disorders such as major depressive disorder (MDD) [40], post-traumatic distress syndrome (PTSD) [41], and traumatic brain injury [41], as well as some diseases with high levels of inflammation—such as arthritis, multiple sclerosis (MS), systemic lupus erythematosus (SLE) and bronchial allergic inflammation [42,43,44,45]—are known to demonstrate disturbed signaling of NTs or Trk receptor.

### 2.2. BDNF-Val66Met Polymorphism

The discovery of a genetic polymorphism within the BDNF gene (rs6265), which causes a valine (Val) to methionine (Met) substitution at codon 66 (Val66Met) in the prodomain of BDNF [46], has thoroughly demonstrated the importance of BDNF in regulating cognitive function and normal brain homeostasis.

The first description of the effects of this polymorphism on memory performance and hippocampal function came more than 10 years ago [46]. The Val66Met substitution reduces secreted BDNF due to abnormal intracellular trafficking [46], which in humans results in decreased hippocampal activity during memory processing [47]. Since the initial discovery of the effects of the BDNF-Val66Met polymorphism on memory formation, a substantial number of articles have been published on the subject. Effects of BDNF-Val66Met on reduced memory performance have been described in preclinical AD [48], MCI [49], and in presymptomatic and symptomatic familial AD [50,51]. The effects of Val66Met in familial AD are not limited to worsened cognition; they can also be seen in lower hippocampal volume, increased total tau and phospho-tau levels [50].

Additionally, a higher amyloid load in combination with Val66Met leads to more rapid cognitive decline in preclinical AD [48,52] and to increased hippocampal vulnerability [53]. It has been demonstrated that Val66Met and APOE4 gene polymorphisms work in concert to increase the amyloid load and in some cases, to more rapid disease progression [54,55,56]. The effects of Val66Met are not only restricted to pathological situations since an effect on memory in healthy older adults has also been observed [54,57,58]. Recently, it was reported that individuals who carry both the Val66Met allele and a polymorphism (rs6347) in the dopamine transporter (DAT) gene showed increased amyloid pathology and greater neurodegeneration [59]. Thus, the presence of the BDNF-Val66Met polymorphism seems to lower the brain’s resilience, and if combined with other insults—such as genetic variants including APOE4 or DAT, amyloid burden or high age—may manifest as a more rapid deterioration of cognitive function. In stark contrast to the effects of the BDNF-Val66Met polymorphism on cognitive function, there have been contradictory findings regarding the impact of the polymorphism on neuropsychiatric diseases, including the lack of correlation with age and the onset of mood disorders [60].

### 2.3. Cellular Signaling of Trk Receptors

**Figure 3:**
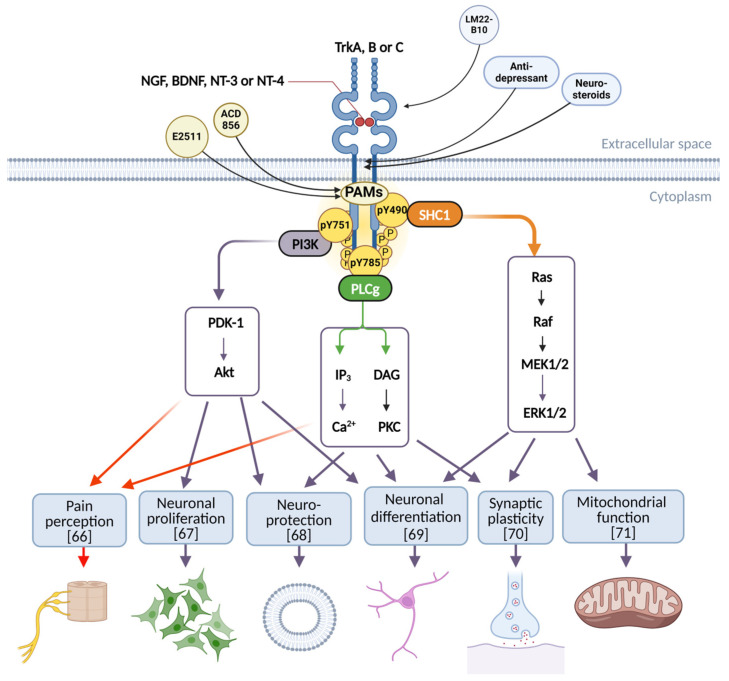
Schematic representation of Trk signaling pathways. The arrows on the upper part indicate suggested sites for interaction between small-molecule modulators and Trk receptors. Phosphotyrosine residues on TrkA are highlighted in yellow circles. Some phosphotyrosine residues are numbered according to the amino acid sequence of TrkA and their interaction with adaptor proteins SHC1, PI3K, and PLCγ are indicated. Functional outcomes are depicted in light blue boxes with citations in square brackets. The figure was created with BioRender.com (https://biorender.com, accessed on 16 June 2024).

At least three major intracellular signaling pathway are involved in the canonical route of Trk receptors (Figure 3). These pathways are activated upon ligand binding and autophosphorylation of tyrosine (Y) 674/675 that governs the catalytic activity of the kinase activity. Following activation of TrkA, phosphorylation of Y490, Y751, and Y785 takes place, leading to a direct interaction of adaptor proteins with the receptor [61,62,63]. For instance, the site of interaction for Src homology domain containing 1 (SHC1) protein on the TrkA receptor is at phospho(p)-Y490, the site for phosphatidylinositol kinase 3 (PI3K) is at pY751, and the site for phospholipase C gamma (PLCγ) is at pY785 (Figure 3). The interaction between the receptor and adaptor proteins can lead to a cascade of downstream events involving calcium mobilization, and activation of several signaling proteins including extracellular regulated kinase (ERK) 1/2, protein kinase B (PKB, aka AKT), protein kinase C (PKC), and downstream transcription factors [63,64,65]. The intracellular signaling downstream of Trk receptors eventually leads to a panel of functional outcomes including, but not limited to, increased pain sensation [66], proliferation or cell survival [67], neuroprotection [68], differentiation [69], increased synaptic plasticity [70], and improved mitochondrial function [71] (Figure 3). The multiple functional effects are most likely explained by the several potential events that occur following NT ligand binding. TrkA levels and function are regulated not only by phosphorylation, but also by ubiquitination [65] and lipids [72]. Interestingly, a point mutation in the transmembrane domain of TrkA (V432E) has been demonstrated to selectively inhibit NGF-induced phosphorylation of ERK1/2 but not the phosphorylation of SHC-proteins, suggesting that downstream signaling of TrkA can be modified selectively by a structural change of the transmembrane domain of TrkA [73]. Results from studies employing phospho-proteome approaches identified more than 700 proteins as downstream targets for TrkA, of which some were specific for their interaction with either Y490 or Y785 or independent of both, suggesting an additional mechanism for interaction [35,74].

The complexity of Trk signaling is evident, especially since there are six additional tyrosine residues in addition to Y490, Y674/675, Y751, and Y785 of TrkA which also could be phosphorylated and participate in regulating downstream events (Figure 3). Only a few investigations have addressed these additional tyrosine residues in detail [75].

The combined effects of genetic deletions or overexpression of different neurotrophins or their receptors demonstrate the complexity and importance of NTs and their Trk receptors in the development and maintenance of both central and peripheral nervous systems. The complexity of the signaling pathways in both spatial and temporal manners suggest that any therapeutics targeting for this system preferably needs to have some pathway selectivity.

### 2.4. Processing of Pro-Neurotrophins

Neurotrophins are produced as C-terminal precursor proteins containing a pre-peptide governing its secretion. The pre-peptide is cleaved off already at the endoplasmic reticulum whereas the pro-form enters the Golgi, where the pro-form can influence sorting, intracellular trafficking, or is re-distributed into secretory vesicles [76]. Two domains in pro-forms, with a conserved amino acid sequence between the different neurotrophins, are most likely contributing to the main functions of the pro-forms [77]. As pro-NGF enters the Golgi network, it can be cleaved into mature neurotrophins or, alternatively, secreted as pro-forms [78]. Intracellular processing of pro-neurotrophins is dependent on endoproteases such as protease convertases (PC), including furin and PCs [79]. Secreted pro-neurotrophins are processed by extracellular matrix proteases to produce the mature forms in a regulated cascade-like manner, as exemplified for pro-NGF [80]. The proteins involved in the cascade of extracellular processing involve tissue plasminogen activator, plasminogen, plasmin, and matrix metalloproteinases (MMPs) including MMP-9 [67].

Given the large number of diseases where neurotrophins or their receptors have been demonstrated to play a role [49,68,81], in combination with the number of affected patients and the current limitation in treatment options [1,2,82], pharmacological interventions of these diseases with molecules targeting the NTs or their receptors are likely to have a large impact on patients’ quality of life as well as on societal health economics. Thus, efforts to identify novel therapeutics targeting the neurotrophin pathways are warranted [83].

## 3. Physiological and Pathological Role of Neurotrophins

Given the fundamental roles of NTs and Trk receptors, the major hurdle to overcome when targeting the NT pathways with novel small molecules is to develop therapeutics that target and normalize the dysfunctional mechanisms, without interfering with the normal neuronal function of NTs, both in the central and peripheral nervous system as well as in non-neuronal cells. Genetic deletion of NGF or TrkA as well as embryonal immunosuppression of NGF leads to severe neuropathies including reductions in trigeminal ganglia, superior cervical ganglia, and a selective loss of certain nociceptive dorsal root ganglia neurons [78,84,85,86]. BDNF and TrkB are well known to be involved in long-term potentiation (LTP) and learning [64]. Targeted disruption of TrkB leads to neuronal deficiencies in both the central and peripheral nervous system, including trigeminal and dorsal root ganglia [87,88]. Likewise, mice lacking BDNF or mice that are heterozygous for BDNF deletion have been extensively studied and some of the pathological findings include reduced learning in an age-dependent manner [89,90]; reduction in the number of neurons in the dorsal root, trigeminal or vestibular ganglions [88]; sensory deficits due to loss of peripheral sensory neurons [91]; respiratory dysfunction [92]; and weight gain [93]. The effects of the BDNF or TrkB genotype on weight gain or obesity have also been described in humans [90,94,95,96]. Interestingly, loss of BDNF did not affect sympathetic ganglia [88], suggesting that the effect is selective for NGF/TrkA. In line with these findings, it was demonstrated that embryonal and postnatal formation of superior cervical ganglia are dependent on TrkA signaling and that the receptor is important to sustain axonal growth, whereas the TrkC receptor was not essential for sympathetic neurons during embryogenesis or postnatal development [97].

Functional TrkC-deficient mice lack projections to spinal cord motor neurons and exhibit movement and axon impairments in the dorsal root ganglia [95,96,98], which suggests a role for TrkC in proprioception. Interestingly, mice lacking both TrkC and NT-3 have fewer oligodendrocyte progenitor cells and deficiencies in other glial cells, including astrocytes [99]. Additionally, NT-3 has been shown to be involved in neurogenesis and LTP in a subset of hippocampal neurons as well as cognitive function [100].

BDNF transgenic animals bearing a BDNF/aCaMKII promoter construct [101] show a chronic 2-3-fold overexpression of BDNF in the forebrain and deficits in learning and memory [102] in otherwise healthy animals, suggesting that excessive BDNF/TrkB signaling in normal young animals may be connected to reduced cognitive function. Interestingly, BDNF is overexpressed in R6/1 mice using the same BDNF/aCaMKII promoter construct, a model for Huntington’s disease, and BDNF-transgenes were essentially found to be devoid of pathological phenotypes [103]. Yet additional studies exploring overexpression of BDNF identified, amongst other findings, increased dendritic arborization and dendritic length in the dentate gyrus [104], a phenotype with increased myelination in the peripheral nervous system whereas spinal AAV-mediated BDNF overexpression was shown to result in an analgesic effect in a model of neuropathic pain [105]. However, there are conflicting reports of the role of BDNF in pain perceptions with several reports suggesting pro-nociceptive effects [104,105,106].

The main functions of neurotrophins and their receptors are diverse and most likely dependent on their spatial distribution and temporal activation patterns. Briefly, the physiological role of NGF is known to be intimately involved in cholinergic function and survival of cholinergic cells in the basal forebrain [107] and in survival and function of sympathetic ganglia [87]. NGF plays an essential role in pain perception [66], which is a normal part of the body’s defense system to avoid tissue injury and to promote healing. However, chronic inflammation, neuropathic pain or other chronic pain states can be deleterious and reduce quality of life for patients. NGF/TrkA contributes to increased pain perception in these chronic pain states, and thus, NGF plays a role in the pathobiology of pain perception [108]. BDNF plays a fundamental role in normal hippocampal function, cognition, and synaptic plasticity [109] and it has been demonstrated that hippocampal LTP is dependent on TrkB-mediated activation of the PLCγ-pathway [110]. Additionally, the important role of BDNF in trophic support can be exemplified by the TrkB-dependent maintenance of prefrontal network circuitry by interneurons [111]. The dependence on BDNF observed for some nerve cells makes them vulnerable to pathological reductions in levels of BDNF. Reduced levels of NGF and/or BDNF can thus lead to pathological situations such as reduced synaptic function, reduced neurotrophic support, and cognitive decline. Reduced levels of neurotrophins have been reported in various situations such as in several neurodegenerative diseases [50,109,112], aging [113,114], and in neuropsychiatric disorders [70,115]. In fact, several antidepressant drugs have been shown to increase the levels of BDNF in serum [116]. The exact mechanism behind the increased levels of BDNF in serum upon treatment with antidepressants is not clear and warrants further research, especially since there are conflicting reports in this area indicating genetic associations of BDNF-Val66Met that are dependent on ethnicity [60].

Considering that neurotrophins regulate cell survival, growth of tumors is one obvious pathological condition which could be characterized by exacerbation of neurotrophin or Trk signaling. This phenomenon is clearly exemplified by Trk fusion protein-driven solid malignancies where the intracellular domain of Trk receptors is fused to different extracellular proteins by gene re-arrangements, leading to an oncogenic constitutively active kinase [117,118].

## 4. Past, Present, and Future Treatment Paradigms of Neurotrophins and Trk Receptors

Several ways to administer NGF into the brain of patients have been evaluated including stereotactic infusion [119], intraventricular infusion [120], intranasal administration [121], implantation of autologous NGF-producing fibroblasts [122], encapsulated cell biodelivery [123], and adeno-associated viral (AAV) delivery of NGF in a clinical phase 1 [124] and phase 2 [125] trial. Some of these approaches have demonstrated beneficial effects, e.g., on CSF cholinergic markers [126] or an increase in FDG-PET [122], but also the reporting of pain as a side effect of the injected NGF. BDNF has been delivered to patients with ALS [127] or diabetic polyneuropathy [128] using subcutaneous injections. Interestingly, one ongoing clinical trial with adeno-associated virus (AAV) delivery of BDNF into the brain will address the effects on delivery of BDNF on neurodegeneration in patients with mild cognitive impairment (MCI) or early AD (NCT05040217). NT-3 treatment in animal models of Charcot–Marie–Tooth type 1A (CMT1A) and administration by subcutaneous injections in patients has demonstrated a beneficial effect on thin myelinated nerve fibers, suggesting a regenerative effect [129]. Later on, an improved way of administering NT-3 to animals by means of AAV was reported also for NT-3 [130].

Improved ways of administering neurotrophins, such as AAV-mediated delivery, might pave the way for new treatment regimens for neurodegenerative diseases. In addition, therapeutics aimed at increasing the levels or the effects of neurotrophins are likely to have a pharmacological and clinical meaningful effect in diseases characterized by reduced neurotrophic signaling.

### 4.1. Small-Molecule Positive Allosteric Modulators of Trk Receptors

Allosteric modulators bind to a site spatially distinct from the endogenous ligand binding site, i.e., the orthosteric binding site [131]. Allosteric modulation has attracted much attention in recent years with examples of molecules targeting different protein classes ranging from ion channels [132], GPCRs [133], nuclear hormone receptors, and receptor tyrosine kinases (RTKs) [131]. Although much of the focus on allosteric regulation of RTKs, and especially on Trk receptors, has been on identifying negative allosteric modulators or allosteric inhibitors of TrkA [134,135,136], there are now recent reports of positive allosteric modulators of Trk receptors [137,138]. Administration of small-molecule positive allosteric modulators of Trk receptors is a more attractive approach than administration of NTs themselves, synthetic receptor agonists or partial agonists, due to their ease of administration, lack of target-related side effects of agonists—such as increased pain sensation—and spatial selectivity. In contrast to an agonist, an allosteric modulator achieves spatial selectivity by modulating the receptor signaling only where ligand–receptor interaction occurs, rather than the widespread receptor activation by a pure agonist. One additional advantage of allosteric modulators could be to potentially induce biased signaling of the receptor, thereby affecting specific intracellular pathways [131]. Positive modulatory mechanisms are likely to obtain the desired therapeutic effect while minimizing side effects.

Fine tuning of Trk receptors by positive modulation is a way to improve neuronal function more specifically, and hence improve the neural network and its connectivity. Positive allosteric modulators of Trks are likely to compensate for the lower levels of neurotrophins observed in AD [112,139] and to normalize the NT-dependent neural network without suffering from the risk of over-activating the receptors. The development of positive modulators of receptor tyrosine kinases have long been hampered by a low degree of druggable binding sites, a general lack of structural information on parts of the receptors such as the transmembrane and juxtamembrane regions, and the complexity of Trk receptor signaling. However, in recent years, major progress has been made in understanding additional mechanisms of existing drugs as modulators of Trk signaling [140,141,142], as well as major breakthroughs in medicinal chemistry efforts leading to the identification of small-molecule positive allosteric modulators of Trk receptors [137,138,143,144].

### 4.2. Previously Described Modulators of Trk Receptors

Several molecules have previously been reported to have a modulatory or agonistic effect on Trk signaling including natural products such as gambogic amide [145], deoxygedunin [146], 7,8-dihydroxyflavone [147], small-molecule peptidomimetics such as tavilermide (also known as MIM-D3) [148], and the more recently described molecule C1 [149]. Additional molecules described as acting on TrkA or TrkB include the tricyclic antidepressant amitriptyline and the neurosteroid dehydroepiandrosterone [150], which act as the starting point for BNN27 [151] and other close analogs. Several agonistic TrkB antibodies have been identified and reviewed elsewhere [152]. Further development of compounds such as 7,8-dihydroxyflavone [147], BNN27 [151,153], LM22A-4 [154], and LM22B-10 [140] has led to molecules with improved properties such as CF_3_CN [155], ENT-A011 [156], ENT-A013 [157], and PTX-BD10-2 [158,159]. Efforts to synthesize dual-acting molecules able to activate both TrkB and 5-hydroytrypatmine receptor 4 (5-HT_4_) has led to the discovery of ENT-C232, a molecule able to activate both TrkB and 5-HT_4_ [160]. However, there are reports describing difficulties with reproducing earlier data and several investigators have reported a lack of observational Trk receptor activation of certain compounds [161,162,163] as well as difficulties in identifying reliable drug candidates [163]. This suggests that assessment of neurotrophic activity of small molecules in in vitro assays should be evaluated using a relevant model for the proposed mechanism of action, or that hits from cell-based assays employing recombinant cell lines should be confirmed by a series of orthogonal assays to verify the activity of the molecules on Trk receptors [137]. One explanation for the lack of effects with compounds such as 7,8-DHF or LM22A-4 on Trk receptors using recombinant cell lines such as the Cellsensor® or PathHunter® assays could be due to the complex downstream cellular signaling of Trk receptors, as demonstrated in Figure 3. Moreover, the degree or pattern of phosphorylation of different tyrosine residues on the receptors may govern the functional outcome. Additionally, the complexity of the mechanism of action for some molecules such as 7,8-DHF—which has been described as having radical-trapping antioxidant properties [164,165]—or other flavones such as formononetin [166], should be taken into account when evaluating results in relation to any TrkB-activating properties in more complex assays or models.

Independent of the contradictory report for LM22A-4, the TrkB/TrkC agonist LM22B-10 and its optimized variant PTX BD10-2 have demonstrated effects in several models [140,158,159]. LM22B-10 was originally identified by an in silico screen and described as a TrkB/C receptor co-activator [140], able to bind to both TrkB or TrkC with an EC50 of approximately 700–800 nM and to displace both BDNF from TrkB and NT-3 from TrkC, suggesting that the molecule interacts with the same binding site as the neurotrophins or that it can allosterically displace the natural ligands while activating the receptor by binding to a different site. It was also demonstrated that LM22B-10 promotes neurite outgrowth, increases spine density [140], and prevents cholinergic dysfunction in a mouse model of AD [159]. BNN27 and its optimized variants are described as acting as agonists of TrkA and TrkB [153,157,167] but BNN27 has also been reported to bind to p75NTR, the pan-neurotrophin receptor belonging to the tumor necrosis family of receptors (TNFR) [151]. Unfortunately, none of the above-described molecules has yet reached clinical development, except for one molecule, the p75NTR-targeting molecule LM11A-31 [168]. The molecule was safe and well tolerated during a 26-week randomized, placebo-controlled, double-blinded phase 2a clinical trial in patients with mild-to-moderate AD. Although there were no significant effects of drug treatment on cognition, several exploratory markers including magnetic resonance imaging, fluorodeoxyglucose positron-emission tomography, and cerebrospinal fluid biomarkers pointed in the direction of reduced disease progression [168].

There have been, up until now, a limited number of compounds described as positive allosteric modulators of Trk receptors, and not acting as agonists or partial agonists. In an elegant series of experiments, Castrén et al. have demonstrated that different classes of antidepressants and psychedelics bind to the TrkB receptor. It was first described that different classes of antidepressant drugs such as fluoxetine, imipramine, and ketamine enhance phosphorylation of TrkB at Y816 (corresponding to Y785 of TrkA) and increase the interaction between TrkB and PLCγ1 [141]. A putative binding site was identified in the transmembrane domain between two TrkB dimers by docking simulations. Amino acid residues identified by modeling as being of importance in the interaction of antidepressants with TrkB were mutated to investigate their role. The V433F mutation was shown to reduce the binding of antidepressants to a large extent, thus confirming the results from the modeling. Interestingly, these data are in line with previously reported findings that the transmembrane region of Trk receptors could have a profound effect on the intracellular signaling pathway [73]. The mechanism of action for antidepressants and their effect on TrkB was described as an allosteric facilitation [141] and this was supported by the fact that BDNF did not displace fluoxetine from TrkB, supporting the notion of two different binding sites.

A second study found that psychedelics, such as lysergic acid diethylamide (LSD) and psilocybin, also bind to TrkB, at a site overlapping with the binding site for antidepressants [141]. Interestingly, psychedelics as well as antidepressants promote the interaction between TrkB and PLCγ1, implying that antidepressants and psychedelics have very similar mechanisms of action on TrkB. Additionally, psychedelics and antidepressants seem to share an allosteric modulatory mechanism of action, as it was shown that psychedelics do not act as agonists, but rather are dependent on endogenous BDNF.

Further, in this study, the antidepressant effects seen for SSRIs, ketamine or psychedelics were independent of 5HT2A [142]. Addition of antidepressants or psychedelics to cells or animals elicited a number of functional outcomes such as induction of LTP, increased survival of neurons, and facilitated formation of long-term memory, all of which were disrupted by Y433F mutation, thereby verifying the importance of TrkB in the mode of action of antidepressants or psychedelics [141,142]. The pharmacological action of fluoxetine and ketamine on neurotrophin signaling seems to be broad since it has been reported that the compounds also bind to and activate p75NTR [169], thus making previous results somewhat more difficult to interpret.

### 4.3. Novel Small-Molecule Positive Allosteric Modulators of Trk Receptors

At present, two different approaches for developing novel small-molecule positive allosteric modulators of the Trk receptors have been documented. First, Eisai has described a small set of molecules acting as biased positive allosteric modulators of TrkA [170] and has presented both preclinical and clinical data for E2511 at several international conferences [138,144,171]. Second, AlzeCure Pharma AB have identified several triazinetrione-based molecules as positive allosteric modulators of TrkA, TrkB, and TrkC [172,173,174,175,176]. The discovery and development of their clinical candidate, ACD856, is described in a series of scientific articles [137,143,177,178,179]. The two examples above are de novo-developed small-molecule positive allosteric modulators of Trk receptors that have entered clinical trials. Both E2511 and ACD856 have been described as having no or very low agonistic effect on receptor function, but rather exert their action by modulating signaling [137,138,143,144].

### 4.4. E2511, a Selective TrkA-PAM

The discovery activities leading to the identification of E2511 as a TrkA-PAM have, to the best of our knowledge, not been disclosed by Eisai. On the other hand, the company has presented substantial amounts of information in the patent [170] and at conferences [138,144,171]. In 2021, Eisai disclosed that E2511 binds to the intracellular juxtamembrane region with a Kd value of 680 nM, and that the phosphorylation pattern of TrkA in primary septum neurons of rats differed after incubation of cells with a low concentration of NGF in combination with E2511 as compared to NGF only, in such a way that phosphorylation of Y785 was higher with E2511 than with NGF only. The difference was not so obvious for phosphorylation of Y490. However, when using human Tau P301S transgenic mice, the levels of phospho-ERK1/2 and phospho-ERK5 were increased after a single oral administration of E2511. There was no effect on phospho-PLCγ, which counters the results obtained using primary neurons from wild-type mice where there was a large effect on phospho-Y785, suggesting an activation of the PLCγ pathway in wild-type neurons [138]. It was also shown that long-term administration of E2511, once daily for 3 months, led to reinnervation of cholinergic neurons in the medial septum of Tau transgenic mice. It was also demonstrated that E2511 had a positive effect on cholinergic function and increased acetylcholine levels (ACh) in neuronal cultures and in cerebrospinal fluid (CSF) from rats, all in a dose-dependent manner [144]. These changes in ACh correlated with increased choline acetyltransferase-positive cells in medial septum neurons in Tau transgenic mice as judged by immunohistochemical analysis, further supporting the neurotrophic effects of E2511.

Interestingly, administration of E2511 for 8 weeks did not lead to hyperalgesia, nor did a single administration lead to a change in bradykinin receptor B2, transient receptor potential cation channel subfamily V member 1 (TRPV1) or substance P mRNA expression in dorsal root ganglia (DRG) [144], suggesting that E2511 can have neurotrophic and neuroprotective effects without induction of pain behavior. Hence, Eisai referred to E2511 as a biased TrkA-PAM, i.e., a compound that can selectively activate specific downstream TrkA pathways.

### 4.5. ACD856, a Pan-Trk PAM

The second example of systematic drug development of positive allosteric modulators of Trk receptors was described by AlzeCure Pharma AB during 2018–2023 [137,143,172,173,176]. The discovery of ACD856 was preceded by high-throughput screening in 2013 and an extensive lead optimization program leading up to the identification of ACD856, a well-characterized pan-Trk PAM. ACD856 was shown by affinity labeling and surface plasmon resonance experiments to interact with the intracellular domain of TrkA [137]. This interaction manifested as an increase in the efficiency of the kinase activity of the Trk receptor [137], thus resembling an inverse mechanism to type IV non-ATP competitive inhibitors or negative allosteric modulators described for certain kinases, including TrkA. Additional experiments demonstrated that a structurally similar compound facilitated induction of long-term potentiation (LTP) in a manner similar to that of BDNF itself. Furthermore, ACD856 reversed scopolamine- or MK801-induced memory impairment in a manner that was sensitive to inhibition of TrkB and additive to acetylcholine esterase inhibitors such as physostigmine [137]. Furthermore, it was demonstrated that ACD856 could improve three different modalities of memory formation, i.e., encoding, consolidation, and retrieval, suggesting a strong multimodal effect on memory formation. In a model of age-induced memory impairment, using 21-month-old wild-type mice, it was shown that a single administration of ACD856, given prior to a learning task, led to significant improvement of memory retrieval as compared to untreated old mice when the animals were tested 11 d after the learning task. In fact, ACD856-treated 21-month-old animals remembered the learning task as well as young mice (4 months old) [137]. The effects on memory performance suggest that ACD856 can have a clear symptomatic effect in patients with cognitive dysfunction, that is additive to the effects of cholinesterase inhibitors.

In a second paper, ACD856 was demonstrated to have disease-modifying effects in preclinical models and to increase the phosphorylation of TrkB and ERK1/2 [143]. It was reported that ACD856 was neuroprotective against amyloid beta or energy deprivation-induced neurotoxicity in primary neurons and that it could enhance NGF-induced neurite outgrowth in PC12 cells, as well as to increase the levels of SNAP25 in neurites. In primary cortical neurons and the brains of aged animals, ACD856 increased the levels of BDNF itself, suggesting a feed-forward mechanism upon enhancement of TrkB receptor signaling, a mechanism that has previously been described for BDNF itself [180,181]. Effects resembling increased neuronal plasticity were also observed in vivo when mice were treated repeatedly for four to five days and then subjected to either a cognition test or to a depression-like model where a sustained pharmacological effect was seen. Apart from the pro-cognitive effects reported for ACD856, antidepressive effects were also demonstrated [182], remaining for up to seven days after the last administration, again indicative of an effect on neuronal plasticity [143]. Briefly, pan-Trk PAM’s, including ACD856, were shown to have a potent antidepressant-like effect in vivo, comparable to that of fluoxetine or ketamine. After 28 days of repeated administration of ACD856, no desensitizing effects on depression-like behaviors could be demonstrated. Furthermore, administration of ACD856 led to a rapid increase in the levels of serotonin, noradrenalin, and dopamine in the lateral hippocampus as measured by in vivo microdialysis [182].

## 5. Clinical Trials with Modulators of Neurotrophin Signaling for the Treatment of Alzheimer’s Disease

Results from first-in-human single- and multiple-ascending-dose studies were recently reported for E2511 [171] and ACD856 [177,178] and are summarized in Table 1.

Single and multiple doses of E2511 were safe and well tolerated with no dose-dependent serious or severe treatment-emergent adverse events. Plasma pharmacokinetics were dose-proportional over the entire tested dose range of 5–80 mg. The plasma half-life of E2511 was determined to be 3.2 h in the single ascending dose study [171]. Moreover, a deep global proteomic approach to identify putative biomarkers using CSF samples from E2511-treated subjects demonstrated a differential expression pattern of certain proteins after a 2-week treatment period. Pathway analysis showed that axonal and synaptic signaling modules were affected after treatment with E2511 [183], thereby supporting the mechanism of action of E2511 as a compound with disease-modifying potential.

The results for ACD856 from both the single- and multiple-ascending-oral-dose studies in healthy subjects were recently reported [177,178]. There was a rapid absorption of the drug and the exposure in plasma increased proportionally with increasing doses of 1–150 mg in single doses [177]. The plasma half-life was approximately 19 h, suggesting that dosing once daily will be sufficient. In the multiple-ascending-dose study, 10, 30, and 90 mg were given once daily for 7 d. In the multiple-ascending-dose study, a dose-dependent increase in ACD856 in CSF was demonstrated, showing a good blood–brain permeability and demonstrating drug CSF exposure at expected clinically relevant concentrations in the brain. More importantly, in the multiple-ascending-dose study, ACD856 demonstrated dose-dependent effects on quantitative EEG, thereby indicating central target engagement without any reported drug-related adverse events [178].

**Figure 4:**
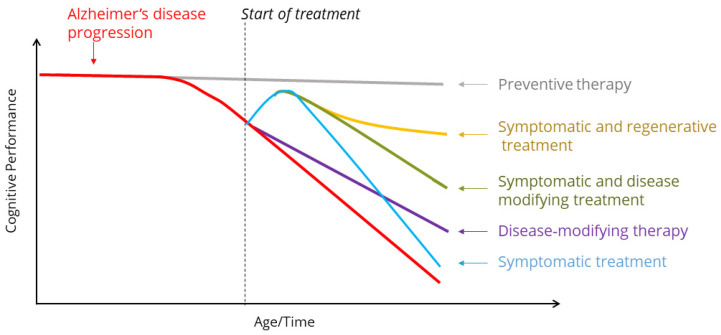
Schematic view of different possible therapeutic modalities on Alzheimer’s disease progression.

The introduction of novel small-molecule positive allosteric modulators of Trk receptors progressing into clinical development is promising and has opened a new avenue for investigational drugs for the treatment of Alzheimer’s disease and other diseases characterized by neurodegeneration, cognitive dysfunction or depression. The existing monoclonal anti-amyloid antibodies have so far been tested in patients with MCI or early AD and have demonstrated disease-modifying effects [1,2,3,4] as depicted in Figure 4. The results from these studies clearly indicate that there is still a need for treatments that address other aspects of the disease, apart from amyloidosis, such as improving the remaining neuronal dysfunction and cognitive disability observed even after clearance of amyloid plaque by anti-amyloid treatment. ACD856, with its cognitive enhancing capabilities, could have complementary effects to both cholinesterase inhibitors and anti-amyloid antibodies, and may have the capacity to improve cognitive function. Considering that ACD856 has a short-term symptomatic effect, and a longer-term effect on neuronal plasticity as well as disease-modifying effects mediated via its neuroprotective and neurorestorative effects, as observed in preclinical models [137,143,182], it is not impossible that the clinical outcome of such effects could be a combination of symptomatic and disease-modifying effects (Figure 4). Since ACD856 in preclinical models has been shown to improve neuro-regenerative effects such as increased neurite outgrowth and increased levels of BDNF, it is tempting to speculate that therapeutics with such outcomes could lead to improved function and increased resilience, allowing the brain to recover from the neurodegenerative effects caused by amyloid plaques, neurofibrillary tangles or neuroinflammation.

**Figure 5:**
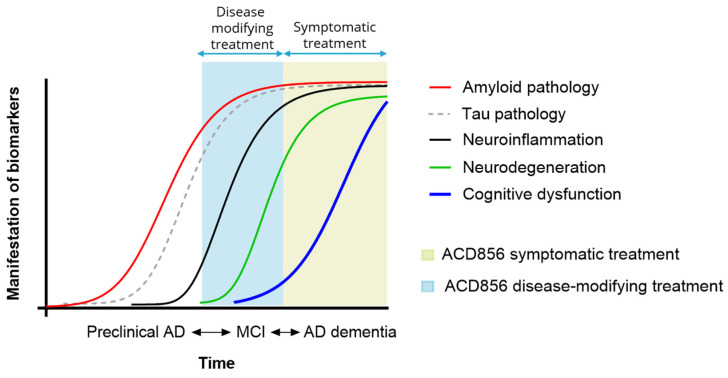
A schematic representation of biomarkers and clinical course of AD and putative temporal treatment options for second-generation symptomatic or disease-modifying treatments.

It is interesting to note that the most desirable effect for AD patients and their families is improvement or restoration of memory function, and second to that, halting the progression of amyloid pathology [184], suggesting that novel therapeutics aiming to increase both cognitive function and to reduce the pathological burden by disease-modifying effects are likely to be received well by multiple stakeholders including patients, caregivers, and societal health systems. Initiation of treatment and the treatment period for molecules acting as modulators of Trk receptors are likely to be dependent on their mechanism of action but will most likely range from mild to early Alzheimer’s disease (Figure 5).

## 6. Discussion

The promising clinical data emerging from the positive allosteric Trk receptor modulator development programs of ACD856 and E2511 are very encouraging and could spearhead the dawn of a second generation of therapeutics that could function as a complement to anti-amyloid antibodies or as a standalone treatment either before or after completion of anti-amyloid treatment. The fact that ACD856 and E2511 in some respects have described similar protective outcomes in preclinical models is reassuring and it gives a validation to positive allosteric modulators of Trk receptors as a mechanism of action to support neurotrophic function. Although there are mechanistic differences between the two compounds, both have demonstrated that targeting the Trk receptors with positive allosteric modulation is a safe and well-tolerated approach for future interventional studies in AD. The symptomatic effects observed for ACD856 are something that has been sought after for a long time in order to complement the existing symptomatic treatments such as the cholinesterase inhibitors.

**Figure 6:**
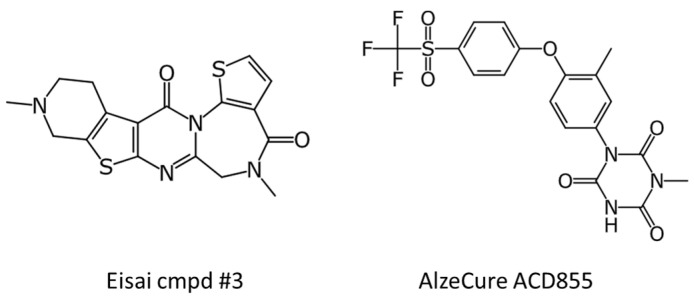
Chemical structure of compounds disclosed in patent applications of scientific journals.

One limitation with this review is the lack of chemical structures disclosed by Eisai for E2511 and by AlzeCure Pharma for ACD856. One can, however, find structures in the publicly available patents or patent applications submitted by the two companies. In the patent application submitted in 2018 by Eisai (US10239889B1), they disclose a limited number of compounds of which the most potent compound (compound #3) is pictured in Figure 6. AlzeCure Pharma disclosed structures in their patent applications during 2017–2019 as well as the structure of ACD855, the predecessor of ACD856 [137]. The structure of ACD855 is shown in Figure 6, demonstrating that the compound belongs to a class of compounds termed triazinetriones. ACD855, also known as ponazuril, was described as an approved veterinary medicine, thus indicating that the molecule is safe and well tolerated in animals [137].

It should also be noted that there are conflicting reports on the involvement of neurotrophins and their receptors in different diseases or in the use of neurotrophins as biomarkers in certain pathological conditions. One such example is depression, where there are conflicting reports on the role of BDNF as a biomarker [185] or on the role of the BDNF-Val66Met polymorphism in major depressive disorder where differences between Caucasian and Asian populations have been reported [186].

## 7. Conclusions

The advancements during recent years in the identification and clinical development of allosteric modulators of neurotrophin signaling is remarkable. There are currently three different modulators with different mechanisms of action in clinical development targeting neurodegenerative diseases such as AD. Tolerability and safety of compounds targeting the neurotrophins or Trk receptors are essential to avoid unwanted side effects. The lack of reported adverse events for E2511 and ACD856 during preclinical development and in clinical trials is very promising, especially when considering that such molecules will likely need to be administered to patients over an extended period. The potential disease-modifying effects observed for E2511 and ACD856 are also encouraging, especially in the light of the beneficial additional symptomatic effects observed for ACD856. The results with these two molecules suggest that they could function well as standalone or add-on therapies to anti-amyloid treatments in the future.

## 8. Future Directions 

Targeted therapeutics using small-molecule allosteric modulators of Trk receptors that in a biased manner activate important intracellular pathways could be key in future treatment of AD and other diseases characterized by disturbed neurotrophin signaling. The clinical results for E2511 and ACD856 warrant further development, with future research likely focusing on interventional studies in appropriate patient populations, presumably in patients with MCI or early AD. Other diseases apart from AD that could benefit from increased neurotrophin signaling include Parkinson’s disease [187], Huntington’s disease [188], SLE [43], MS [43,44], neuropsychiatric diseases [70], TBI [41,121], CMT1A [129,130], and inflammatory diseases such as osteoarthritis [30,42], but also other types of diseases with reduced neurotrophic support such as acquired hearing loss [189].

Future studies should also be focused on increasing the molecular understanding of the biased signaling observed for Trk modulators, increasing the understanding of the role of neurotrophins in different compartments of the body in relation to certain diseases, and clarifying the role of their genetics, such as the BDNF-Val66Met polymorphism.

## Figures and Tables

**Table 1:** Summary of clinical phase 1 studies of ACD856 and E2511.

ACD856	Single dose											Multiple dose—7 d					
	Placebo	ACD856 (mg)								Total		Placebo	ACD856 (mg)				Total
		1	3	10	20	40	75	150	Total				10	30	90	Total	
N	14	6	6	6	6	6	6	6	42	56		6	6	6	6	18	24
Sex (M/F)	11/3	5/1	5/1	5/1	5/1	5/1	6/0	6/0	37/5			5/1	6/0	5/1	5/1	16/2	21/3
Age, years (mean[SD])	43.9 (12.8)	38.5 (13)	39.2 (17)	35.3 (11)	33.0 (13)	44.0 (16)	32 (6)	30 (6)	36	40.5		46.3 (14)	29.5 (7.1)	41.3 (16)	45 (15)	38.6	40.0
Adverse events (AE)	No dose-dependent, serious or severe treatment-related AEs. Most common AE was headache due to lumbar punctures.																
Safety	No significant findings in vital signs, ECG, labs, EEG (MAD only), or physical examinations were reported																

E2511	Single dose											Multiple dose—14 d					
	Placebo	E2511 (mg)								Total		Placebo	E2511 (mg)				Total
		5	10	20	40	80			Total				10	30	90	Total	
N	10	6	6	6	6	6			30	40		6	6	6	6	18	24
Sex (M/F)	6/4	3/3	4/2	5/1	4/2	4/2			20/10	26/14		6/0	4/2	3/3	4/2	11/7	17/7
Age, years (mean[SD])	34 (9)	38 (13)	40 (13)	34 (8)	35 (9)	36 (9)			36 (10)	36 (10)		45 (6)	27 (4)	36 (9)	32 (5)	32 (7)	35 (8)
Adverse events (AE)	No dose-dependent, serious or severe treatment-related AEs. Most common AEs was headache due to lumbar punctures.																
Safety	No significant findings in vital signs, ECG, labs, EEG, or physical examinations were reported																

## Data Availability

Not applicable.
